# Immunohistochemical Investigation of Mutant BRAF V600E in Common Pigmented Skin Neoplasms, Study on a Sample of Iranian Patients

**DOI:** 10.30699/IJP.14.1.8

**Published:** 2019-12-27

**Authors:** Maryam Ghasemi, Laleh Vahedi Larijani, Omid Emadian, Jamshid Yazdani, Amad Sajadianfar, Saeid Abediankenari

**Affiliations:** 1 *Associate Professor, Dept. of Pathology, Immunogenetics Research Center, Mazandaran University of Medical Sciences, Sari, Iran*; 2 *Assistant Professor, Mazandaran University of Medical Sciences, Sari, Iran*; 3 *Associate Professor, Mazandaran University of Medical Sciences, Sari, Iran*; 4 *Associate Professor, Dept. of Health, Mazandaran University of Medical Sciences, Sari, Iran*; 5 *Resident, Mazandaran University of Medical Sciences, Sari, Iran*; 6 *Professor, Immunogenetics Research Center, Mazandaran University of Medical Sciences, Sari, Iran*

**Keywords:** RAF gene, Mutation, Immunohistochemistry, Pigmented skin neoplasm

## Abstract

**Background & Objective:**

This study was designed for the first time for the detec- tion of mutant BRAF V600E and its correlation with clinicophathologic features in a sample of Iranian patients with pathologically proved pigmented skin neoplasms.

**Methods:**

82 paraffin-embedded blocks, including melanocytic nevi, malignant melanoma, Basel cell carcinoma, and squamous cell carcinoma were evaluated for BRAF V600E expression by immunohistochemistry in the patients admitted to Ibn Sina Hospital, in the city of Sari, Mazandaran province, North of Iran. The evaluation of immunohistochemical staining was performed by two of the authoring pathologists, and staining intensity was graded from negative (0), weak (1+), moderate (2+) to strong (3+). If twenty percent (or greater) of the tumor cells showed modest to strong cytoplasmic immunoreactivity (score 3+), the neoplasm was considered positive for this tumor marker.

**Results:**

Among 82 studied patients, 12 cases (60%) of the malignant melanoma group revealed a high intensity of immunostaining for BRAF V600E, while a signifi- cant expression of this marker did not occur in the other investigated skin neoplasm. A great relation between BRAF (V600E) expression and the histologic type of skin cancer was noted. No significant relationship with other parameters such as gender, age, and the grade differentiation of the non-melanoma skin cancer was found. BRAF V600E was weakly correlated with the Clark level of cutaneous malignant melanoma.

**Conclusion:**

This data provided further evidence for the strong role of the BRAF V600E mutation in the development of cutaneous malignant melanoma, compared to non-melanoma skin cancers in the North of Iran. We advised future studies to evaluate the beneficial effects of anti-BRAF V600E target therapy on the Iranian melanoma patient who harbors this marker by way of immunostaining tumor tissue.

## Introduction

Malignant skin neoplasms are the most common skin malignancies in Iran and the world. According to stud- ies in Iran, it includes almost 14.6% of cancers (1,2,3). Despite significant advances in medical science, these neoplasms continue to be a great burden on healthcare (4-8) in Iran. SCCs, BCCs, and melanomas are among the most important skin cancers. Noteworthy BCCs and SCCs can show pigmentation, and some other pigmented skin neoplasms may have borderline manifestations that simulate malignant melanomas, the most aggressive kind of skin cancer (7,8). A variety of tumor markers that are useful in differentiating benign and malignant neo- plasms and different types of cancers have been studied. The BRAF oncogene has been considered as one of themost important markers that play a role in the pathogenesis of tumors including thyroid, ovarian, and colorectal carcinoma, as well as malignant melanoma (9-13). The BRAF oncogene transmits growth signals to protein kinases on the RAS oncogenic pathway. The most important and frequent mutation of BRAF is V600E, which results in the substitution of the valine amino acid by glutamic acid at position 600, thereby dysregulating the activation map of the kinase/ERK signaling pathway, leading finally to melanoma genesis (14,15). Therefore, it can be a good subject for studies to differentiate benign and malignant skin neoplasms, thus predicting tumor behavior and planning specific target therapy by new drugs such as vemurafenib against BRAF V600E (12).

There are some molecular and immunostaining-based studies, both in in vitro and in vivo settings, with in- consistent results regarding the prevalence, clinical implications and correlation with pathologic and prognostic variables of BRAF V600E in skin neoplasms 20 (13). The frequency of the BRAF V600E mutation was reported as ranging from 6.4% (15) to as high as 70.1% (16) in the skin melanomas of patients around the world. We were encouraged for the first time to investigate the expression of mutant BRAF V600E in a sample of Iranian patients in the north of Iran that had suffered from pigmented skin neoplasms. We chose the available immu- nohistochemical method for the detection of this oncogene due to its sensitivity, specificity, simplicity and cost effectiveness (21,22)

## Materials and Methods

This study examined the expression of mutant BRAF V600E in benign and malignant pigmented skin neo- plasms from tissue samples from the pathology department of Ibn Sina Hospital in Sari, Iran. According to primary estimates, 82 samples were included.

Samples included patients with pathologically proved pigmented skin cancer, who had previously underwent excisional biopsies. Also, Paraffin-embedded blocks from people without skin cancer, however possessing pig- mented nevi, were evaluated.

Clinicopathological features such as gender, age, histologic type, the grade of non-melanoma tumor differentia- tion, and the Clark level of malignant melanomas were included. We examined papillary thyroid carcinomas and normal skin tissue paraffin-embedded blocks as positive and negative controls, respectively.

Immunohistochemical staining was performed on samples with 4 µm thickness, which were cut by use of a device. Then, samples were put on special slide and heated for 60 minutes via hot air oven at 60°C. To remove paraffin, xylenol, as well as absolute and 96% ethanol were used, and the slides were rinsed with tap water. After the slides were dried, they were put in a container of 1% oxygenated water and methanol for 10 minutes. They were then transferred to the target solution and remained in the autoclave under 1.5 atm. pressure, and were afterwards left there until they reached room temperature. After rinsing with tap water and a wash buffer, tissue borders were identified using a pen. Then, the samples were left in humid room. The surface of the samples was covered using a diagnostic kit of Moues Anti-human BRAF, V600E Monoclonal Antibody Clone VE1 (cata- logue number E19290) from the Spring Biosciences company. Then, samples were put in an “Envision” at room temperature for 60 minutes. After two steps of rinsing, DAB wash buffer solution was poured onto the slides, and after 1 to 2 minutes of color change, the slides were again put in a wash buffer solution for 2 minutes. Slides were washed with still water after using Mayer’s haematoxylin, and were fixed in Xylenol. They were then mounted with entellan glue. Finally, the slides were pathologically assessed by light microscope (Nikon) by two experts, who evaluated them for intensity cytoplasmic staining, as well as percentage of immunoreactive cells, compared to the above-mentioned positive and negative controls. The immunoreactive cells revealed brown cy- toplasmic discoloration whose intensity was graded from negative (0), mild (1+), moderate (2+) to strong (3+). The BRAF (V600E) intensity of cytoplasmic staining was in accordance with the percentage of immunoreac-tive cells reported in the 4 scores used by the kit’s manufacturing company.

1. (0): no color –negative.

2. (1+): less than 5% - weak and dispersed.

3. (2+): 5% to 20% - mild cytoplasmic.

4. (3+): more than 20% - medium to strong, cytoplasmic around the nucleus.

Scores less than 3+ were considered to have low staining or negative, and scores of 3+ were considered to have high staining or positive for BRAF V600E.

In the next step, the data was analyzed using statistical software SPSS (IBM SPSS Statistics 23.0.0). To de- scribe the results briefly, quantitative data like age was explained as mean ± deviation from standard, and quali- tative data were explained in grouped data. A t test was used to compare patient age, and the chi square test was used to compare sexes in the two groups. Finally, statistical logistic regression was used to compare differences in BRAF V600E expression. *P*<0.05 was considered statistically significant.

## Results

82 patients including 49 males and 33 females were examined. The participants’ clinicopathological features are shown in table 1. Patient mean age was 50.2 years (confidence Interval 95 %, 46.53-54.03) regardless of patient sex. There was no significant relation between BRAF (V600E) expression and the parameters of sex and age (*P*>0.05). Expressions of BRAF V600E and staining intensity were compared between groups (table 2).

Due to the randomized selection of data, all of the fourteen BCC cases were found to have well-differentiated tumors, but only 9 cases of 23 SCC cases had well-differentiated tumors, while 11 cases had moderately dif- ferentiated tumors, and three of them exhibited poor differentiation. No relation between SCC tumor grade of SCC and the expression of BRAF V600E was identified (*P*=0.152). Furthermore the differentiation of BCC was constant, and its relation with BRAF V600E could not be calculated. A weak positive relationship between the Clark level of the malignant melanoma and the expression of this tumor marker was found (*P*=0.04)

**Figure 1:**
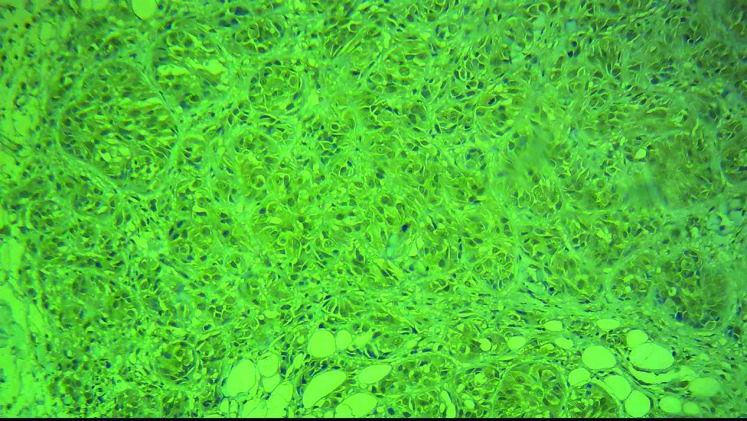
Melanoma cells showed diffuse cytoplasmic BRAF V600E immunoreactivity (score3+, X200 magnified)

**Figure 2:**
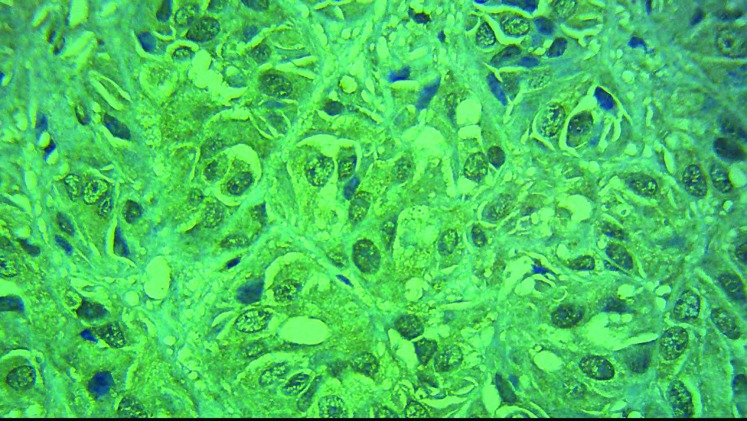
Melanoma cells showed diffuse modest cytoplasmic BRAF V600E immunoreactivity (score3+) (X1400magnified

**Figure. 3:**
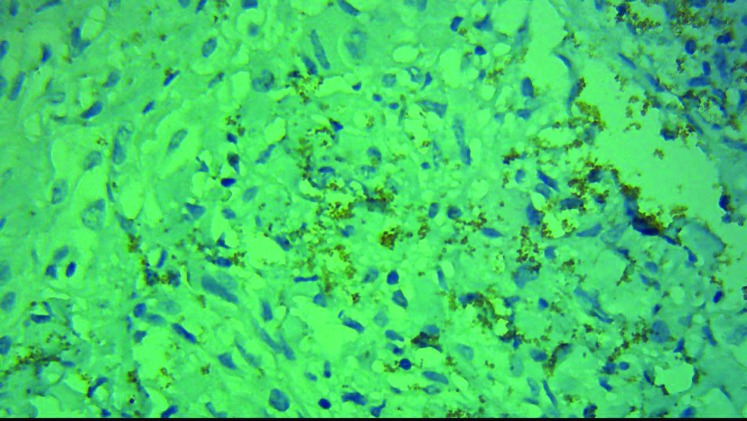
Melanoma cells showed dispersed week cytoplasmic BRAF V600E immunoreactivity (score1+) (X400magnified)

**Figure 4:**
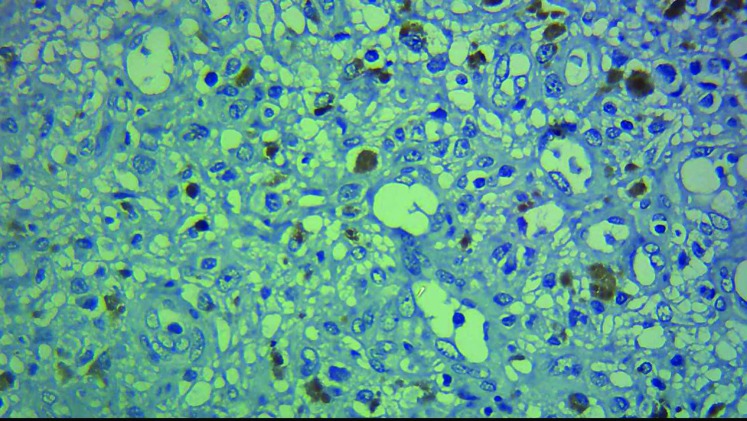
Melanoma dells showed focal mild cytoplasmic BRAF V600eE imunoreactivity(score2+)(X400magnified)

All of the malignant skin melanoma cases showed immunoreactivity with BRAF V600E in immunohistochem- ical staining, 60% showed high intensity (score +3, Figure 1, 2) and the remaining 40% showed low intensity (score 1+ and 2+) (Figure 3, 4). 48% of the pigmented nevi, 43.5% of the SCC and 21.4% of the BCC exhib- ited low staining intensity, and none of them showed high staining intensity. According to the definition in the “materials and methods” section, 60% of the malignant melanoma group expressed BRAF V600E, and thus a significant difference in intensity of BRAF V600E staining between melanoma and non-melanoma skin cancers was found (*P*<0.001). In our study, as shown in Fig. 3, normal tissue around the tumor and papillary thyroid carcinoma paraffin-embedded blocks were used as negative and positive controls respectively.

**Figure 5:**
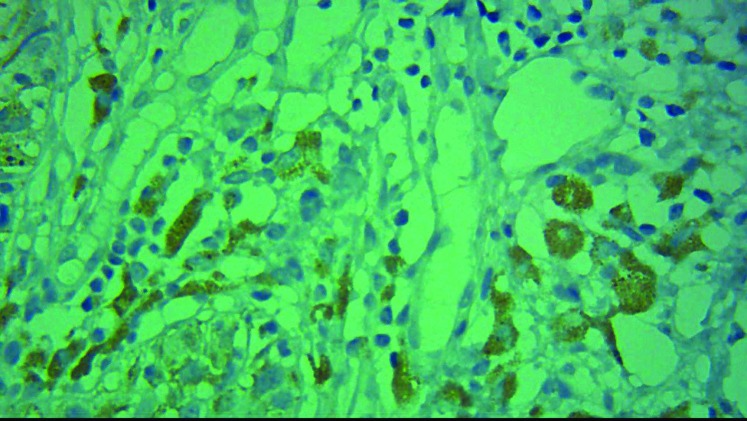
Note the false positive staining of BRAF V600E marker in the cytoplasm of the melanophages cells in the nevus

As shown in Fig.5, melanophages revealed false positive reactions that should not be considered as expressions of mutant BRAF V600E.

**Table 1:** Clinicopathological features of patients with benign and malignant pigmented skin neoplasms

**Features**		**Frequency**		**Total**	
Age	Less than 50 years old 35% (n=29)	More than 50 years old 65% (n=53)		100% (n=82)	
Sex	Male 59.8%(n=49)	Female 40.2%(n=33)		100% (n=82)	
Histological type of skin tumor	Pigmented nevu s 30.5%(n=25)	Malignant melanoma 24.4% (n=20)	BCC 17.1% (n=14)	SCC 28.0% (n=23)	100% (n=82)

**Table 2:** Comparison of BRAF (V600E) expression and staining intensity in benign and malignant pigmented skin neoplasms

**Intensity of Staining**				**Pathological diagnosis**					
				**Pigmented nevus**		**Malignant melanoma**	**BCC**	**SCC**	N
				13		0	11	13	37
	0	Total percentage		35.1%		0.0%	29.8%	35.17%	100%
		Percentage found to be pathological		52.0%		0.0%	78.6%	56.5%	45.2%
				9		1	2	10	22
	+1	Total percentage (%)		40.9%		4.5%	13.6%	40.9%	100%
		Percent due to pathological diagnosis		36.0%		5.0%	14.2%	43.5%	26.8%
N				3		7	1	0	11
	+2	Total percentage (%)		27.3%		63.7%	9.0%	0.0%	100%
		Percent due to pathological diagnosis		12.0%		35.0%	7.2%	0.0%	13.4%
	+3			0		12	0	0	12
		Total percentage		0.0%		100%	0.0%	0.0%	100%
		Percent due to pathological diagnosis		0.0%		60.0%	0.0%	0.0%	14.6%
			25		20		14	23	82

## Discussion

Skin cancers are considered an important international issue in terms of diagnostic, treatment and follow-up costs in medicine. A search on that matter has led us to understand that no study in our region was conducted on the prevalence of BRAF V600E expression in skin cancers. We conducted this study in order to elucidate the frequency and pattern of the immunohistochemical expression of BRAF V600E among common pigmented skin neoplasms in a sample of Iranian patients living in the north of the country. 60% of the evaluated malignant melanoma patients expressed this mutant BRAF more commonly than populations in other reports (13-20). Patients who were sampled by Zepeda Lopez had a frequency of 6.4% (15), while patients of Yi-Hue Liao rep- resented 14.3% (17), and patients of Emilia Hugdall 35% (14). Inumaru et. al. detected BRAF V600E mutations in 70.1% of melanoma patients (16). Our study, similarly to other previous studies (8,19), showed no significant association between the BRAF gene mutation and non-melanoma skin cancers. As mentioned above 43.5% of SCC and 21.4% of BCC subjects revealed low intensity staining for BRAF V600E with the immunohistochemi- cal method.

In addition, 48% of investigated pigmented nevi represented low intensity staining for this marker. These findings should be confirmed by molecular analysis of the BRA V600 mutation.

Laura Held studied proliferative activity, chromosomal defects, and tumor mutation via gene hybridization, and assessed BRAF in differentiating blue nevi from melanomas. Eventually she did not find any relation between mutations, the amount of mitoses (*P*=0.03) and patients’ outcomes (23).

Douglas Fullen et. al. studied BRAF mutations in Spitzoid melanocytic lesions, and borderline lesions suspect- ed to be Spitzoid. Their results show that a small subgroup of Spitz nevi with atypia harbored BRAF mutations; therefore, assessing BRAF mutations cannot differentiate all Spitz nevi from Spitzoid melanomas (24). A larger sample including pigmented nevi and other skin cancers, which count as challenges of differential diagnosis of malignant melanomas, were investigated in our study.

Goel et. al. studied the formation of melanocytic hyperplasia and melanomas in transgenic mice by inducing this mutation. They found that BRAF mutations cause the progression of nevi to overt melanoma (25). In our study, which was based on a human model and immunohistochemical staining, there was a high frequency of BRAF V600E expression in malignant skin melanoma with significant differences between them and examined nevi (*P*<0.001).

Long e.t al. compared the sensitivity and specificity of the immunohistochemical staining of BRAF V600E in paraffin-embedded samples of patients with stage 4 malignant melanoma, which required the molecular confir- mation of this mutation for target therapy. A sensitivity of 97% and specificity of 98% were observed in their work (21). In fact, our results showed that the IHC method was valuable in identifying the presence of mutant BRAF V600E as a tumor marker in Iranian melanoma patients.

Fisher et. al. studied IHC scoring in challenging cases of malignant melanoma, which required the assessment pf the BRAF V600E mutation for target therapy. In 57% of negative cases from COBAS molecular tests, IHC confirmed the results (22). In a clinical trial, Paola Ascierto et. al. examined the role of the BRAF mutation in melanoma by using the first specific inhibitor of the BRAF V600E mutation, called Vemurafenib. Results showed a 63% reduction in mortality risk and a 74% decrease in tumor progression (26). In our study, a signifi- cant association between the immunohistochemical expression of BRAF V600E and malignant skin melanomas was noted. Also, strong attributes in differentiating benign and malignant neoplastic lesions including pigmented nevi, pigmented BCC and SCC from melanoma were confirmed. Although the molecular method is preferred in the screening of malignant melanoma for target therapy, here, immunohistochemistry is suggested due to its simplicity, availability and the fact that it is less time-consuming. With the advancement of new target therapy against BRAF V600E, this method may be useful in assessing the response to this modality of treatment, reduc- ing costs and complications of the treatment in Iranian patients who don’t express this marker.
